# Growth Mixture Modeling of Loneliness Trajectories in Older Adults: The Role of Cognitive Function

**DOI:** 10.1093/geroni/igaf122.2188

**Published:** 2025-12-31

**Authors:** Elnaz Abaei, Peter Martin

**Affiliations:** Iowa State University, Ames, Iowa, United States; Iowa State University, Ames, Iowa, United States

## Abstract

Loneliness is a growing public health concern among older adults in the United States, with significant implications for cognitive function. This study utilized conditional growth mixture modeling (GMM-CV) to investigate the longitudinal trajectories of loneliness and how cognitive function (measured by immediate word recall, delayed word recall, and serial 7s) predicted these trajectories. Data were drawn from a sample of 3,944 older adults (Mage = 68.74) using eight waves of the Health and Retirement Study (HRS) data from 2006–2020. Data from every two years were pooled to increase the sample size and create four time points. GMM-CV in Mplus identified three distinct loneliness trajectories best fit the Data: LL= -25,495.269, BIC=51,214.097, SSABIC=51,128.303, Entropy=0.814, Adj. LMR-LRT (.52)=245.630, BLRT(.51)=255.518. The three identified classes were: Class 1: Low-Stable Loneliness (75.6%), Class 2: High-Decreasing Loneliness (13.1%), and Class 3: Moderate-Increasing Loneliness (11.3%). The mean group differences revealed significant group differences in immediate word recall at time 1 (F = 3.19, p=.04) and time 4 (F = 3.9, p=.02), delayed word recall at time 1 (F = 3.31, p=.037) and time 4 (F = 3.11, p=.045), while no significant effect was found for serial 7s at time 1 (F = 2.33, p=.098) and time 4 (F=.068, p=.934). Immediate word recall at time 4 exhibited a significant negative effect on the loneliness slope, indicating that higher cognition scores were associated with a less steep increase in loneliness over time. These findings suggest enhancing cognitive function as a potential intervention to reduce loneliness in later life. Future research should explore underlying mechanisms and moderating factors.

